# The First Nations experience of accessing rheumatology services in a metropolitan hospital: A qualitative study

**DOI:** 10.1111/hex.14049

**Published:** 2024-04-16

**Authors:** Taylor Cullen, Miki Griffith, Arvin Damodaran, Ebony Lewis, Faye McMillan, Anthony Sammel, Rhiana Honeysette, Brett Biles, Kim Beadman, Sally Nathan

**Affiliations:** ^1^ School of Population Health, Faculty of Medicine and Health, UNSW Sydney Australia; ^2^ Department of Rheumatology Prince of Wales Hospital Sydney Australia; ^3^ Aboriginal and Torres Strait Islander Community Services Prince of Wales Hospital Sydney Australia; ^4^ School of Clinical Medicine, Faculty of Medicine and Health, UNSW Sydney Australia; ^5^ School of Public Health University of Technology Sydney Australia

**Keywords:** caregiver engagement, equity, First Nations Australians, integrated care, participation, patient engagement, rheumatology

## Abstract

**Objective:**

First Nations Australians experience a higher burden and severity of Rheumatic Disease with poorer outcomes than the general population. Despite a widely acknowledged need to improve health outcomes, there has been minimal research assessing existing models of care from a First Nations perspective in Australia. The objective of this study was to describe First Nations experiences and barriers and enablers to accessing a hospital‐based adult Rheumatology service in Sydney.

**Methods:**

A qualitative study using semi‐structured interviews was undertaken. Patients who self‐identified as First Nations attending the Prince of Wales Hospital Rheumatology Clinic in 2021 were invited to participate. Interviews were conducted face‐to‐face or by telephone using culturally‐appropriate Yarning methods with an Aboriginal Health Worker (AHW) at the request of participants. Thematic analysis was done in consultation with an Aboriginal Reference Group (ARG).

**Results:**

Four categories, which encapsulated 11 themes were identified. Participants reported barriers to care such as logistics of the referral process, not feeling culturally safe because of uncomfortable clinic environments and health worker behaviours, inadequate cultural support and community perceptions of the specialty. Enabling factors included family member involvement, AHW support and telehealth consultation.

**Conclusion:**

The current model of care perpetuates access challenges for First Nations Australians within rheumatology. Barriers to care include the delayed referral process, limited cultural responsivity in the clinic environment and poor cross‐cultural communication. There is a need for models of care that are co‐designed with First Nations Peoples to address these barriers.

**Patient and Public Contribution:**

Participants were First Nations Australians with lived experience attending the rheumatology clinic. All interviewees were offered the opportunity to review their transcripts to ensure trustworthiness of the data. Preliminary thematic analysis was conducted in partnership with the AHW who has over 20 years experience. Following preliminary coding, a list of themes were presented to the ARG for iterative discussion and refinement. The ARG provided community representation and ensured that First Nations voices were privileged in the analysis. It's intended that the findings of this study will support the upcoming co‐design of a First Nations health service for Rheumatology patients.

## 1 INTRODUCTION

First Nations Australians are the oldest continuous living culture in the world and remain strong and resilient.^1^ However, experience some of the worst health outcomes globally.^2^, ^3^ This stems from dispossession, past policies, racism, discrimination and marginalisation of mainstream health services, which can be attributed to the far‐reaching consequences of colonisation.^4^, ^5^

Rheumatology is a unique specialty dealing with musculoskeletal and complex immune‐related disease often requiring immunosuppressive medication.^6^ Early intervention of rheumatological disease is essential to prevent long‐term poor outcomes and continuity of care relies on a good clinician‐patient relationship.^7^, ^8^, ^9^ First Nations Australians overrepresented in chronic conditions, including rheumatic disease, are, however, less likely to receive specialist care, resulting in poorer health outcomes.^10^, ^11^ For example, a recent study of outpatient specialist clinic access amongst First Nations Australians reported they were 1.5 times more likely than non‐First Nations Australians to be referred to clinic services but less likely to book and attend.^11^ First Nations patients reported barriers to accessing care include transport limitations, inadequate referral pathways, language barriers and a lack of cultural sensitivity from providers and the physical environment.^12^, ^13^ Australia's projected Rheumatologist workforce shortage will likely exacerbate access to specialist care.^14^

Studies from Canada and New Zealand have demonstrated how models of care in Rheumatology can be improved for First Nations peoples. For example, provision of services at First Nations primary care practices, incorporation of First Nations arthritis liaisons, culturally‐adapted communication tools and clinician cultural upskilling.^15^, ^16^, ^17^, ^18^ These models found an overwhelming majority (89%) of participants were satisfied with services and improved disease outcomes.^17^ A recent Australian publication has provided insight into the possible benefit of providing Rheumatology specialist services from an independent community‐based First Nations health service showing increased patient numbers by comparison with hospital‐based services.^19^ Nonetheless, research has found current rheumatology services remain inadequate to meet the needs of First Nations Australians, and there is a paucity of research on the enablers and barriers of using these services from an Australian First Nations perspective.^12^, ^20^ This has implications for how rheumatological health services currently meet the needs of this population. Therefore, we aim to describe First Nations Australians perspectives of a major metropolitan service and the limitations and enabling factors which impact the experience of care.

## 2 PATIENTS AND METHODS

### 2.1 Participants and setting

This study was based in an outpatient rheumatology clinic at Prince of Wales Hospital in Sydney, Australia. Most patients (~75%) are referred via primary care providers (GPs), with the remaining referred through the emergency department (ED) and specialty physicians in the hospital. Costs are covered under the publicly funded health system, Medicare. Referrals are triaged for urgency and there may be wait times for initial appointments. Four rheumatologists, a rheumatology trainee, nurse and three administrative staff service the clinic. First Nations patients may receive assistance from the community Aboriginal Health Unit in the form of Aboriginal Health Worker (AHW) support but this is not standardised. The study authors are Rheumatology healthcare providers and academic researchers, including an AHW (detailed in Supporting Information S1: Table 1). First Nations adults who attended the Rheumatology clinic in 2021 and had a diagnosis of a musculoskeletal or autoimmune condition were considered eligible to participate. Ethics approval was granted through the Aboriginal Health and Medical Research Council (AHMRC 1828/21) and Hospital Ethics Council (HREC 2021/ETH11659).

### 2.2 Data collection

Patients were informed about the study by their treating clinician. Those who expressed interest in the study were contacted by the research team who provided further explanation of the study before obtaining consent using a verbal script. They were informed that the study team aimed to translate the findings into a new model of rheumatology care that is culturally appropriate and beneficial to First Nations peoples. Participants were offered the option to participate in‐person or via telephone and for an AHW to be present. Demographic characteristics of referral source and diagnosis were collected from the patient's medical records. The participants were offered the opportunity to review their interview transcript for accuracy.

Using an Indigenist research approach we aimed to draw on First Nations ways of knowing, being and doing through semi‐structured interviews guiding a ‘research topic yarn’ as defined by Bessarab et al.^21^ The interviews were preceded by ‘social yarning’ which helped familiarise the participant with the interviewers. The transition to research topic yarn was signposted by a verbal consenting process and indication that the recording would begin. A purpose‐built interview guide was developed by the lead author (T. C.) with the two Aboriginal healthcare worker authors (M. G., R. H.) (Supporting Information S1: Table 2). Research Topic Yarning was used to generate focussed discussion around participant experiences of rheumatology services and was administered by T. C. and M. G.^21^ Interviews ranged from 13 to 45 min and were audio‐recorded and transcribed.

### 2.3 Data analysis

A reflexive thematic analysis of interview transcripts was undertaken using an inductive approach.^22^, ^23^, ^24^, ^25^ Familiarisation and preliminary coding was conducted by TC using NVivo12 as a data management tool to code and compare data across the interviews. A second researcher (M. G.) with a First Nations perspective reviewed the preliminary coding, and a list of themes were jointly developed (T. C., M. G.). The overall study team were involved in iterative discussions of the possible themes and interpretations were reviewed and verified with the Aboriginal Reference Group (ARG) to ensure that First Nations voices were privileged in the analysis. A worked example of how this information was presented for review and processed is included in Supporting Information S1: Table 3 for transparency.

## 3 RESULTS

In 2021 consecutive patients who attended the rheumatology clinic and were identified as Aboriginal or Torres Strait Islander were invited to participate. Two patients declined, 18 consented, and 16 (4 males and 12 females) aged between 23 and 77 participated. Over half (56%) were aged 40+ years. Half were referred to the outpatient clinic by their GP, the remainder from ED and specialists. Five patients had a family history of Rheumatic Disease. The majority were referred for inflammatory disease (*n* = 11), and osteoarthritis was the most common noninflammatory disease (*n* = 4).

There were 19 preliminary themes generated, these were reviewed by the ARG and divided into four categories and 11 themes which describe First Nations peoples experiences in accessing a hospital‐based adult Rheumatology service.

### 3.1 Category 1: Access to care

#### 3.1.1 Theme A: Referral pathways and waiting times lead to confusion and negative first impressions

Nearly all participants reflected that wait times to access rheumatology clinics are a barrier to care. Participants reported feeling they wanted to give up because there was a large delay between GP referral and their specialist appointment.

‘my doctor kept doing referrals. They just get pushed back…’ ‘through your local GP you've got no hope’. (Female 40–59 years)

One exception was those who presented via the ED. Two participants reported that visiting the ED was a positive experience and facilitated outpatient appointments.

if I can't get into the GP, go to emergency and see someone there and they will get someone from the department that can come and hopefully fix what you need. (Male <40 years)

In some cases, it was reported the GP referral did not clearly outline the reason for patient referrals and led to experiences of frustration at the initial consultation and confusion surrounding what treatments the rheumatology service could offer.

when I actually came, they sort of said to me, oh we can't help you, we need to refer you back to the infectious diseases clinic… it's hard to obviously read everyone's files and I'm not sure what my referral said from my GP either, but that was just a waste of a half a day. (Female < 40 years)

#### 3.1.2 Theme B: Transport is key to service access

Most participants described how they arrive to hospital and commented on transportation challenges including cost, inconvenience and how this could exacerbate symptoms.

Some people don't drive and they have to get a bus to get up there and they're in pain too, so yeah, it does make it hard. (Female > 60 years)

#### 3.1.3 Theme C: Telehealth services can act as an enabler to accessing care but quality may suffer

A number of participants had telehealth appointments during the COVID pandemic. Some found this helpful in improving access, whilst others felt that the quality of care provided was decreased compared with face‐to‐face consultations. In‐person consults were felt to be particularly important for initial assessment.

she was doing consultations through zoom calls. It was really hard for her, you know push here, push here and trying to hold her phone and I've got kids jumping on me at the same time. (Male <40 years)

I think for some people it's a lot easier to wait at home for a phone call than coming all the way into hospital. (Female <40 years)

### 3.2 Category 2: Cultural responsivity of services

#### 3.2.1 Theme A: Cultural safety and the clinic environment

Several participants reported experiencing a lack of cultural safety, usually in the waiting room rather than during health consultations. This was experienced as feeling looked down upon or unwelcome. However, some participants commented that this was also possibly related to stressors of administrative staff dealing with large volumes of patients.

you don't really need people at the counter looking down at you. (Female 40–59 years)

you're just in a big waiting room. No one talks to you. You feel like cattle. (Female 40–59 years)

For some, these impressions were ameliorated with recognition of their culture through actions such as the addition of familiar artwork and imagery in the waiting room.

there is a picture of my Aunty in one of the hallways that leads from one of the NAIDOC things not long ago, so there is definitely people from the community around. (Male <40 years)

#### 3.2.2 Theme B: Preference for service delivery at an aboriginal community centre

Based on past experiences, a number of participants suggested that delivering services from a culturally safe Aboriginal Community Health Service would improve access.

It's more welcoming [the Aboriginal‐led clinic] because everyone is treated the same out there. (Female 40–59 years)

it would be easier if they had a clinic down at our, at La Perouse, a medical clinic, it would be good if the doctors came out there. (Female >60 years)

#### 3.2.3 Theme C: Rheumatology services were seen to cater to an older population

Most younger patients (<40 years) felt there was a community impression that Rheumatology was a service for older people. This stereotyping of the service acted as a barrier to seeking care.

I know I am one of the younger patients that uses the clinic, so that is one of the biggest difficulties I have, not anything you do, just overcoming the personal issues with using a clinic that is mainly for older people. (Female <40 years)

For some younger participants, community perceptions that rheumatology catered to older adults only was linked to a number of self‐perceived negative emotions. For these participants, accessing services was linked to feeling ‘shame’ and ‘embarrassment’. These feelings were also a barrier for seeking emotional support from others as illustrated by one participant.

I think the shame of it as well, you didn't want to say it to anyone, it was such a crazy thing to not be able to really walk or run probably for like a year or two. (Male <40 years)

#### 3.2.4 Theme D: The role of effective communication and the importance of feeling listened to

Multiple participants reported that feeling heard by the health professionals was an important factor to acknowledge the social and emotional wellbeing components of their care.

no one is listening to what I'm saying or taking into consideration my value or anything so it's really killing me. (Female <40 years)

There was often miscommunication due to the use of medical jargon or the complexity of information. Finding common ground to establish effective communication was seen as central to good care. Concepts which were generally well understood by participants were ‘arthritis’ and ‘pain’ and this information tended to come from the community rather than health providers. There was a perceived need for culturally adapted health information to enhance understanding for some participants.

language, you just need to get people who are going to sit there and be able to talk to us and tell us what's going on and what the big issue is with us. Cause even I speak English I need someone there to tell me the proper way: ‘this is your diagnosis, this is what you have to go and do this is what you’. (Female <40 years)

… you can't give them a medical explanation, you may as well just fly in the wind—but I think the pamphlets are good, especially like black fella cartoons which I think works, because Aboriginal people do relate to that very much. (Male 40–59 years)

#### 3.2.5 Theme E: AHWs can help bridge the gap between biomedical and holistic models of care

Participants who had support from AHWs felt this was beneficial. AHWs have a deep cultural understanding and detailed knowledge of the health system. They were reported to assist with logistics and could act as a patient advocate facilitating cross‐cultural communication.

You need a one‐on‐one person [AHW] that's going to really support the Indigenous people and sit down and tell them, you know look, this is what's happening this is what's going on, this is what you need to do, do you need any help? This is what we can provide for you and this is how we go about stuff. (Female <40 years)

### 3.3 Category 3: Understanding rheumatic disease

#### 3.3.1 Theme A: The role of a rheumatologist and the nature of rheumatic conditions were not well understood

Most participants were not aware of the role of a rheumatologist before clinic attendance. This fed into a sense of uncertainty about management options and the necessity of follow‐up. When asked about their illness, patients referenced pain as the central problem. They occasionally ascribed a medical label to their symptoms, for example, ‘lupus’ without explanation of the disease aetiology.

‘no idea… I feel like I haven't even pronounced it properly’ ‘until I came here I wouldn't have known that this is who I come to see about gout’. (Male <40 years)

managing pain, I guess that's the easiest way to describe it. (Female >60 years)

the lupus is more or less to do with the bones and I don't think much can be done about it. (Female >60 years)

When people did describe their health conditions the knowledge was most commonly extrapolated from community experiences rather than through physician or health service provided information. One exception was among participants who had a background of working in health.

… speaking to my aunty…she's saying that one of my cousins would have flare ups from strawberries. (Male <40 years)

I'm a registered nurse by trade, so I've got a pretty good medical background, so I'm pretty much aware of you know all of it. (Male 40–59 years)

Some participants were interested in having more information on their condition. Provision of education was thought to require spending time discussing the condition during clinic appointments, using visual resources to assist with understanding complex medical information and ensuring consistency in physician messages.

I would like to know more, and it's also passing it onto other indigenous people, you know like the need to find out more about your body… because we come from a background of really not questioning enough. (Female 60+ years)

pamphlets specifically would be very helpful… rheumatology, my God it's a difficult one to get your head around. (Male 40–59 years)

### 3.4 Category 4: The role of community in disease management

#### 3.4.1 Theme A: Rheumatic disease contributes to a disruption of community roles and responsibilities

Multiple participants reported fatigue and pain impacting their function and contributing to difficulty fulfilling their usual community roles. This caused feelings of guilt and disappointment which were seen as isolating. Some also reported difficulty communicating symptoms to others who weren't visible.

I usually go & babysit or go do food shopping… a lot of it requires walking & so a lot of it I can't do anymore so I have had to change to giving money to help people get movers or really planning out, like I'm going to babysit you on this day, no matter how I feel… So it's equated into a lot of cancelled plans for my nieces & nephews which has really frustrated them because they don't understand that I'm actually sick. (Female <40 years)

Conversely, certain patients delayed seeking care because they prioritised community responsibilities or the wellbeing of others over their own health. This specifically applied to women holding a matriarch role in the family.

there's always another medical issue and a more pressing medical issue you know, anyway, I don't give enough attention to my own medical needs I suppose to be honest. (Female >60 years)

#### 3.4.2 Theme B: The culture of kinship is integral to First Nations communities and provides support for those affected by rheumatic disease

Participants who had family members with rheumatic diagnoses reported that this was a major supportive factor in their care. Participants explained that having family with similar issues helped validate their experience, negotiate health systems and manage treatment expectations. This culture of supporting and sharing experiences was perceived as a great source of strength in managing disease.

it kind of helps because mum and dad both have it and my little sister has very similar pain too, so when we are all in pain, we all kind of talk and we realise it's the same kind of thing, so that makes it more real because sometimes I just feel like my body is hurting for no reason. (Female <40 years)

## 4 DISCUSSION

This study provides the first assessment of specialist rheumatology care through a First Nations Australians lens. It complements research conducted across other specialties including oncology, pain and renal medicine, supporting the centrality of communication as a key barrier to First Nations patients accessing care and the important role of community and family in managing chronic conditions.^26^, ^27^, ^28^ It also describes perspectives on rheumatology‐specific issues including challenges explaining complex immune diseases expectations of care, and the stereotyping of rheumatology as a specialty for older people.

Nonlife threatening referrals carry wait times, however many rheumatological conditions cause pain and disability in the short term, with damaging social consequences and poorer outcomes from delayed care.^29^ Through theme 1A participants reported that presenting via ED allowed for streamlined access to specialist clinics. The setting of anticipated workforce shortages, wait times and the referral process for rheumatology services are growing concerns.^14^ The referral pathway requires additional support to bridge the gap between referral and clinic review to prevent overburdening ED.^30^, ^31^ Participants reported that AHWs provided support in navigating health services and facilitating cross‐cultural communication in the theme 2E. In other research, AHWs have reported that this is achieved through careful development of trust, empathy and respect.^32^ Offering AHW support at the time of GP referral may build more support into the system.^32^ AHWs are uniquely positioned to bridge cross‐cultural communication and facilitate access to care.

Through the theme, 2D participants explained that communication between community members and their clinicians and administrative staff was identified as another barrier to care. Participants reported feeling unheard in consultations and requested the assistance of a cultural ‘translator’ to help understand their diagnoses and management. There has been increased emphasis placed on the cultural safety of health professionals ensuring they have the necessary communication skills to practice culturally‐safe care.^33^ Developing these skills requires clinician upskilling through educational programs run by First Nations peoples which have been delivered in Canadian settings and more recently Australia.^34^, ^35^ In Australia, there is evidence that ‘yarning’ is a helpful strategy for health professionals to reduce communication barriers with First Nations Peoples.^36^, ^37^

Participants suggested that culturally appropriate educational resources, including pamphlets and infographics, could also help bridge communication. Australia's peak arthritis association has recently published dedicated education resources developed with First Nations peoples.^38^ It is critical to embed such resources in the training of Rheumatology staff to assist with establishing culturally safe practices.

Aboriginal Community Controlled Health Services (ACCHSs) are an effective First Nations‐led model which improves health outcomes in Primary Care.^39^ Engagement with these services could benefit rheumatology practice.^19^, ^39^, ^40^, ^41^ The success of ACCHSs is based on their ability to address the clinical, social and cultural determinants of health leading to a more holistic approach in line with the Aboriginal definition of health.^39^, ^42^ In theme 2B participants reported hospital environments included uncomfortable waiting rooms, transport challenges and negative staff attitudes which acted as barriers to care. Offering services at community clinics run by First Nations organisations may overcome these barriers by offering a culturally familiar setting.^32^ Evidence shows that urban First Nations outreach clinics improve outcomes for Canadian First Nations rheumatology patients.^15^, ^17^ Similarly, a recent Australian publication demonstrated that providing rheumatology care from a First Nations‐led service increased attendance rates compared to existing hospital clinics.^19^

Similar to the findings in this study in theme 2C, the stereotype that rheumatic disease primarily affects older adults has been documented in other populations.^43^ Though this study represents a small sample of participants, further investigation should be done to clarify if the perspective is held more broadly, such perceptions could negatively impact patient care through delaying access to care. Overcoming these perspectives requires education, the First Nations‐led education for vaccination during COVID is one example of how this can be achieved.^44^ Furthermore, in theme 4B several participants reported an important role for family members affected by rheumatic diagnoses because they understood the symptoms and helped with navigating health services. Involvement of family in healthcare delivery may be a further way to support patients receiving care.^45^

Key strengths of the study include its indigenist research methodology and collaboration with First Nations people in all aspects of design, data collection and interpretation. In particular, the involvement of the AHW who had an intimate understanding of the local health service and community and provided a unique contextual background to the interviews. This helped build rapport with participants and supported effective open communication in a culturally safe manner.^46^

There are limitations to this study, including the small cohort. Fortunately, almost all the patients approached to participate in this study consented and the sample of participants was diverse in age, gender and diagnosis. Thus, it was felt sufficient to represent key issues being experienced by First Nations patients at this clinic. Nevertheless, First Nations Australians are a diverse group and as such this research cannot be generalised to all First Nations Australians. Furthermore, in recruiting from the clinic there is a bias towards participants who are willing to attend hospital services. It's notable that only 50% of participants were GP referred by comparison with 75% from the general clinic population. This may suggest there are patients in community not referred for services and in turn there is a group of people experiencing barriers at a pre‐clinic stage which we may not have identified.

## 5 CONCLUSION

This study documented the perspective of First Nations Australians on rheumatic disease care in an urban clinic setting. It is clear there are ongoing barriers to service access especially related to referral pathways, cultural responsivity of the clinic setting and cross‐cultural communication challenges. Engaging clinicians in delivering culturally safe practices revising the clinic environment so that it is culturally safe, incorporating culturally tailored education resources and involvement of AHWs and families in care are likely to improve the experience and in turn outcomes of First Nations Australian patients in rheumatology. Future research should focus on First Nations Australians led design of models of care to address these issues.

## AUTHOR CONTRIBUTIONS

**Taylor Cullen**: Conceptualisation; investigation; writing—original draft; methodology; validation; writing—review and editing; visualisation; formal analysis; project administration; data curation; funding acquisition. **Miki Griffith**: Conceptualization; investigation; writing—review and editing; formal analysis; data curation; validation. **Arvin Damodaran**: Writing—review and editing; supervision; methodology; data curation. **Ebony Lewis**: Writing—review and editing; supervision; data curation; methodology. **Faye McMillan**: Methodology; investigation; supervision; formal analysis; data curation; validation. **Anthony Sammel**: Writing—review and editing; investigation; supervision. **Rhiana Honeysette**: Data curation; formal analysis. **Brett Biles**: Formal analysis; data curation; validation. **Kim Beadman**: Formal analysis; data curation; validation. **Sally Nathan**: Supervision; investigation; methodology; writing—review and editing; formal analysis; project administration; data curation.

## CONFLICT OF INTEREST STATEMENT

The authors declare no conflict of interest.

## Supporting information

Supporting information.

## Data Availability

The data that support the findings of this study are available on request from the corresponding author. The data are not publicly available due to privacy or ethical restrictions.
