# Supplementary material for: The First Nations experience of accessing rheumatology services in a metropolitan hospital: A qualitative study
Source: Health Expect. 2024 Apr 16;27(2):e14049. doi: 10.1111/hex.14049 (PMC11019439; doi:10.1111/hex.14049)
Supplement: Supplementary file 1 — Supporting information. [file HEX-27-e14049-s001.docx]

## Supplementary Tables

### Table 1 – Research team

| Researcher | Experience |
| --- | --- |
| TC (Non-First Nations) | Rheumatology Advanced Trainee with a Master of Public Health and Tropical Medicine and several years experience training and working with communities in Far Northern Australia where a large proportion of the population identify as First Nations peoples. |
| MG (First Nations) | A proud Bundjalung woman working and living on Bidjigal and Gadigal land with 20 years experience working as an Aboriginal Health Worker at Prince of Wales and the associated health network. She holds a bachelor in health science majoring in Aboriginal Health and Community Development. |
| RH (First Nations) | A proud Ngiyampaa woman from Wiradjuri Country in the Riverina who grew up on Bidjigal Country and has been working in Aboriginal Health since 2012. Currently employed as an Aboriginal Health Worker at Prince of Wales Hospital. |
| BB (First Nations) | Brett is a Murrawarri man from Brewarrina. He has been living on Wiradjuri country for the last 20 years. He holds a Bachelor of Physiotherapy, a Masters in Indigenous Health and a PhD. He is currently Associate Dean Indigenous with Medicine and Health and a Senior Scientia Lecturer at UNSW. |
| KB (First Nations) | Coordination of the Gujaga Community reference panel with Wodi Wodi heritage and experience working on several Aboriginal Health projects.  She holds a Bachelor of Psychological Science, Bachelor of Nursing and post graduate studies in Drug and Alcohol nursing and forensic mental health nursing. |
| EL (First Nations) | A Wiradjuri woman working and living on Bidjigal land. She holds a Bachelor of Nursing and a Master of International Public Health. She is a lecturer and course convenor of the Aboriginal Health and Wellbeing Specialisation at the School of Population Health at UNSW Sydney. |
| FM (First Nations) | A proud Wiradjuri yinaa (woman) originally from Trangie, NSW, now living and working between rural/regional and urban NSW. She has over 20 years of experience in the Higher Education Sector and over 30 years in the health sector with and extensive list of accolades including being made a Fellow of the Pharmaceutical Society of Australia  the PSA Australian Pharmacist of the Year in 2022, the Australian Medal (AM) in the General Division of the Order of Australia for "significant service to Indigenous Mental Health, and to tertiary education" in the 2021 Queen's Birthday Honours List, 2019 NSW Aboriginal Woman of the year, Deputy National Rural Health Commissioner for Allied Health and Indigenous Health. |
| AD (Non-First Nations) | Rheumatologist and academic researcher with an intimate knowledge of the site at which the study was conducted. Director of Teaching for the School of Clinical Medicine at UNSW Sydney. |
| TS (Non-First Nations) | Rheumatologist and academic researcher with an intimate knowledge of the site at which the study was conducted. |
| SN (Non-First Nations) | Associate Professor and public health social scientist whose research is characterised by active partnerships with non-government organisations, including the Aboriginal Community Controlled Health (ACCH) Sector, to improve health equity and opportunities for young people, key groups and communities. She is Convenor of Foundations of Public Health, & Qualitative Research Methods in the postgraduate programs in the School of Population Health, UNSW Sydney. |

### Table 2 – Interview guide

| INTERVIEW GUIDE |
| --- |
| 1. What is your understanding of your illness and how has it affected you or your family? 2. Why do you think you need to come to this clinic? 3. How have you tried to manage your symptoms? 4. What treatments do you understand are available through the hospital clinic or your doctors? 5. What have we done right/wrong in the clinic to support you? 6. What has helped or stopped you from accessing services? How could we improve access? |

### Table 3 – Example of thematic workup with ARG

## Preliminary theme and supportive quotations presented to the ARG following TC and MG initial analysis

### Preliminary theme: Cultural safety begins in the waiting room

The waiting room provides a first impression as to what the clinic experience will be like. Some patients reported feeling ‘different’ or unwelcome. This was experienced this as a combination of direct racism from staff and systematic issues related to the public system trying to cater to such a large volume of people. Recurrent suggestions were made to improve this experience through changing the environment using familiar imagery e.g. family photos or artwork so that people would feel as though they belonged.

| INTERVIEW | QUOTE |
| --- | --- |
| 1 | “there is a picture of my Aunty in one of the hallways that leads from one of the NAIDOC things not long ago, so there is definitely people from the community around” |
| 2 | “you’re just in a big waiting room. No one talks to you. You feel like cattle.”   About La Perouse clinics “It’s more welcoming because everyone is treated the same out there” |
| 4 | “.. always uncomfortable in hospitals, but that’s just general feel, like I’m not supposed to be there”  “sitting in the waiting room is a bit uncomfortable because people look at me and either think I’m not supposed to be there, or they don’t”  “if you ran an aboriginal rheumatoid arthritis clinic, even if it was every 4 months, I would sign up for the Aboriginal one. Not that the workers have to be Aboriginal, just that everybody in there is Aboriginal I would prefer that because I feel more comfortable surrounded by mob than I do around other people, but that is very much a personal thing”  “if it is run out of one of the AMS’s or something like that, although those can be a bit full on & rushed. I would prefer it in the normal setting just like you know, one Friday a month, it’s Aboriginal only or something, if you had enough clients.”  Suggestion for improvement: “making the waiting room a bit more welcoming & inviting, the chairs & stuff, I know it’s clinical, but it feels very clinical, like if you were to have couches & stuff instead of the hard backed chair. I don’t know making the waiting area a bit more comfortable especially because people will be waiting a bit for appointments” |
| 5 | “the clinic I went to .. was an Aboriginal health clinic, so they are really, really good at looking after you.” (in reference to a previous AMS clinic)  “you don’t really need people at the counter looking down at you, going you go and sit over there and they will come out when they’re ready”  “I rock up with a flag t-shirt or something and the doctors are cool with it, but the front staff aren’t that enthusiastic” |
| 8 | “the staff who are, you know, being friendly at the front, they always wrist down on a card and.. it’s always just been easy to go to the clinic”  “I think everything runs smoothly, you know, like it’s fine, starting with the nurses at the front, at reception, they can’t do enough for you sometimes, like with your appointments & they’re lovely girls, & the doctors are fine yeah, I mean I don’t have anything bad or anything to bring up about the clinic because it’s fine” |
| 13 | “Because you know everyone that’s there, so it’s more friendly, cause when you get to the hospital they have different girls on the desks, some are nice, some are not so nice, you know you know everyone who works down here. That’s why it’s better” |

## ARG member feedback

| ARG Member | Feedback | Quote felt to best encapsulate the theme |
| --- | --- | --- |
| 1 | - A fair representation - Imagery is a very important but it’s also important to highlight that it’s not the only thing impacting the way people feel, if systemic racism is present imagery won’t fix that - Cultural issues can be invisible by comparison with other issues, for example physical disability, if somebody wheels in in a wheelchair, we make provision because we need to, because we can see it. This needs to be highlighted. - Clearly the environment overall needs to be a welcoming.. and that's something that I think every Aboriginal person feels - Staff education is very important in ensuring cultural safety | “you’re just in a big waiting room. No one talks to you. You feel like cattle.”   About La Perouse clinics “It’s more welcoming because everyone is treated the same out there” |
| 2 | - Consider that there's a lot of different language around cultural out there, for example; cultural safety, awareness, training, responsivity, cultural competencies. I think you need to be consistent with your language and terminology here. The AHPRA document gives a good definition of what cultural safety is from an Aboriginal perspective. - Cultural safety is fluid. The idea around culture of safety can change, it has to be interpreted in the context of a person or client centred approach. For example, if I'm seeing you in the emergency department, at this acute crisis point and then I come and see you in three months time when I've got my pain under control, my cultural safety requirements and my particular client centric approach is going to be different in those scenarios. - The fundamental aspect of what we're trying to achieve here is to make our workplaces and healthcare systems free of racism | Agree with above |
| 3 | - The daunting clinical environment is a big thing in the hospital - Having artwork does help clients to feel more comfortable, they can create a positive feel before they even interact with staff - Clients will sometimes perceive people being racist rather than just attributing it to the staff member having a bad day, this is informed by cultural experiences and is important for staff to remember. The service isn’t designed to tailor care to the individual in response to this. It is a problem. | “making the waiting room a bit more welcoming & inviting, the chairs & stuff, I know it’s clinical, but it feels very clinical”  “cause once you get in there, you can’t get out, you’ve got to fight to get out” “it’s been like that my whole life, I won’t stay in hospital” |
| 4 | - First impressions are everything and you may not talk to somebody at the clinic for the 1st 5 minutes but you may have already picked up a leaflet or looked at an artwork. - Our experience is informed by going to these clinics with patients and sitting there, really experiencing the environment and the way that staff interaction with the patients. | “like I’m not supposed to be there. I think that is an internalised me thing, it did affect my coming to appointments.”    “That happened to my Aunty up in Cowra, my Aunty went in there spewing up & next minute they give her something & next minute she has a heart attack, she’s only 42, so how does that work out?” |

## Consolidated ARG feedback

| THEME – CULTURAL SAFETY OF SERVICES | KEY REPRESENTATIVE QUOTES |
| --- | --- |
| As has been referenced in the recent national guidelines by AHPRA it is the responsibility of the practitioner to understand the complex history of colonialism in Australia and how this inherently underpins the interactions between First Nations patients and the health system.    Cultural safety begins with first impressions and the addition of familiar artwork and imagery can make the clinic environment more comfortable when combined with other facets of care and staff. Recurrent suggestions were made to improve the clinic experience through changing the environment using familiar imagery e.g. family photos or artwork so that people would feel as though they belonged.  This will not fix systemic issues of racism but goes a small way to starting any consultation in a more comfortable environment and can buffer first impressions of feeling like patients don’t belong within a large institution.    Suggestions   - Education of medical by First Nations people - Use of yarning techniques to communicate education and culture synchronously - Complex trauma feeds into the likelihood of accessing hospital based services – the clinic environment is important and moving the clinic may be helpful | “you don’t really need people at the counter looking down at you”    “you’re just in a big waiting room. No one talks to you. You feel like cattle.”    “making the waiting room a bit more welcoming & inviting, the chairs & stuff, I know it’s clinical, but it feels very clinical”    “like I’m not supposed to be there. I think that is an internalised me thing, it did affect my coming to appointments.”    “cause once you get in there, you can’t get out, you’ve got to fight to get out” “it’s been like that my whole life, I won’t stay in hospital”    “That happened to my Aunty up in Cowra, my Aunty went in there spewing up & next minute they give her something & next minute she has a heart attack, she’s only 42, so how does that work out?” |

## Final theme

Category 2: Cultural responsivity of services

Theme A: Cultural safety and the clinic environment

Several participants reported experiencing a lack of cultural safety, usually in the waiting room rather than during health consultations. This was experienced as feeling looked down upon or unwelcome. However, some participants commented that this was also possibly related to stressors of administrative staff dealing with large volumes of patients.

*“you don’t really need people at the counter looking down at you”(Female 40-59years)
“you’re just in a big waiting room. No one talks to you. You feel like cattle”(Female 40-59years)*

For some, these impressions were ameliorated with recognition of their culture through actions such as the addition of familiar artwork and imagery in the waiting room.

*“there is a picture of my Aunty in one of the hallways that leads from one of the NAIDOC things not long ago, so there is definitely people from the community around”(Male <40years)*
